# Greening or browning? The macro variation and drivers of different vegetation types on the Qinghai-Tibetan Plateau from 2000 to 2021

**DOI:** 10.3389/fpls.2022.1045290

**Published:** 2022-10-26

**Authors:** Huihui Wang, Jinyan Zhan, Chao Wang, Wei Liu, Zheng Yang, Huizi Liu, Chunyue Bai

**Affiliations:** ^1^ State Key Laboratory of Water Environment Simulation, School of Environment, Beijing Normal University, Beijing, China; ^2^ School of Labor Economics, Capital University of Economics and Business, Beijing, China; ^3^ College of Geography and Environment, Shandong Normal University, Jinan, China

**Keywords:** greening, browning, climate change, restoration, MODIS NDVI, Qinghai-Tibetan Plateau (QTP)

## Abstract

Vegetation greenness is one of the main indicators to characterize changes in terrestrial ecosystems. China has implemented a few large-scale ecological restoration programs on the Qinghai-Tibetan Plateau (QTP) to reverse the trend of ecosystem degradation. Although the effectiveness of these programs is beginning to show, the mechanisms of vegetation degradation under climate change and human activities are still controversial. Existing studies have mostly focused on changes in overall vegetation change, with less attention on the drivers of change in different vegetation types. In this study, earth satellite observation records were used to robustly map changes in vegetation greenness on the QTP from 2000 to 2021. The random forest (RF) algorithm was further used to detect the drivers of greenness browning on the QTP as a whole and in seven different vegetation types. The results show that an overall trend of greening in all seven vegetation types on the QTP over a 21-year period. The area of greening was 46.54×10^4^ km^2^, and browning was 5.32×10^4^ km^2^, representing a quarter and 2.86% of the natural vegetation area, respectively. The results of the browning driver analysis show that areas with high altitude, reduced annual precipitation, high intensity of human activity, average annual maximum and average annual minimum precipitation of approximately 500 mm are most susceptible to browning on the QTP. For the seven different vegetation types, their top 6 most important browning drivers and the ranking of drivers differed. DEM and precipitation changes are important drivers of browning for seven vegetation types. These results reflect the latest spatial and temporal dynamics of vegetation on the QTP and highlight the common and characteristic browning drivers of vegetation ecosystems. They provide support for understanding the response of different vegetation to natural and human impacts and for further implementation of site-specific restoration measures.

## Introduction

Terrestrial vegetation communities include forests, shrubs, meadows and grasslands (Franklin et al., 2016). They play a key role in global biogeochemical cycles of carbon, nitrogen, oxygen and water while supporting economic activities, including forestry and grazing, and providing important ecosystem services, such as carbon sequestration, water harvesting, and wind and sand control (Franklin et al., 2016; Deng et al., 2022). As a fundamental component of terrestrial ecosystems, the distribution and condition of vegetation communities often affect the qualities of animal habitats (Stein et al., 2014; Seibold et al., 2019). Therefore, changes in vegetation communities may have important impacts on the response to global climate change, the conservation of biodiversity and the sustainable development of human society (Hansen et al., 2013; Wang et al., 2021). At the national, regional and global levels, a series of ambitious ecosystem restoration targets have been proposed to address multiple major socio-environmental challenges, such as global change and biodiversity loss, and thus improve people’s livelihoods (Strassburg et al., 2020). The United Nations (UN) has declared 2021-2030 the United Nations Decade for Ecosystem Restoration (Aronson et al., 2020). The Bonn Challenge and the New York Declaration on Forests aim to restore 350 million hectares of land globally by 2030 (Chazdon et al., 2016; Verdone and Seidl, 2017; Keenan and Riley, 2018). The United Nations Convention to Combat Desertification and Sustainable Development Goal (SDG) 15 called on the international community to achieve “zero growth in total land degradation” by 2030 (United Nations, 2015). Monitoring and understanding the impacts of natural and human activities on vegetation is fundamental to achieving these goals.

In recent years, the monitoring of vegetation dynamics changes using remote sensing images as a data source has been rapidly developed (Fensholt and Proud, 2012; Yang et al., 2021; Berner and Goetz, 2022). Earth observation satellites have provided decades of image data that can establish long-term spectral vegetation index time series to quantitatively assess regional and even global changes in vegetation greenness (Gao et al., 2020). The normalized difference vegetation index (NDVI) is one of the most widely used spectral vegetation indices and is often used as a measure of the greenness of aboveground vegetation (Pettorelli et al., 2005; Pettorelli et al., 2011). For example, in cities, NDVI was used as neighborhood greenness to study the relationship with the mental health of residents (Liu et al., 2019). From reginal to global scale, researchers have chosen NDVI to indicate the greenness status of vegetation such as forests (Hilker et al., 2014), grasslands (Miao et al., 2021) and tundra (Myers-Smith et al., 2020), etc. An increase in NDVI indicates a greening of the vegetation greenness and, conversely, a decrease in NDVI indicates a browning (Myers-Smith et al., 2020; Wang et al., 2021). Therefore, time series analysis of NDVI can characterize the changes in vegetation over time and thus provide a basis for further exploration of the drivers of changes in vegetation dynamics. NDVI long time series can be constructed from accessible remote sensing data such as Advanced Very High-Resolution Radiometer (AVHRR), Landsat 4/5/7/8/9, and SPOT (Gao et al., 2020). However, in long time series data, the inconsistency of satellite sensors and data introduces a large uncertainty (Peng et al., 2012; Pan et al., 2017; Liu et al., 2021c). The MODIS NDVI dataset used the same sensor data from 2000 to the present. Additionally, the improvement of synthetic data algorithms has improved the ability to monitor the changes in MODIS NDVI products, thus avoiding the problems of sensor degradation and data uncertainty to some extent (Didan et al., 2015; Chen et al., 2020; Liu et al., 2022).

Understanding the drivers and impact thresholds of vegetation change is a key foundation for the effective management of ecosystems (Zhu et al., 2016; Easdale et al., 2018). Climate (Jiang et al., 2017; Li et al., 2021b; Huang et al., 2022), soils (Zhong et al., 2022), topography (Xu et al., 2020; Liu et al., 2022), and human activities (Shi et al., 2021) are considered key drivers of changes in vegetation dynamics. In previous studies, the variability of greening and browning processes in different types of vegetation has been widely reported (Zhu et al., 2016; Pan et al., 2018). For instance, in a study by Peng et al. (2012), the dynamic trends of different vegetation types on the QTP from 1982 to 2013 were found to be clearly different. Research by Yan et al. (2021a), a study in southwest China, reported that the greenness of evergreen needle-leaved forest and grassland among ten vegetation types was mainly controlled by climatic factors. This may result from the inconsistent processes of different vegetation communities in response to environmental changes (Wu et al., 2018). The spatial heterogeneity of natural and anthropogenic drivers has also been considered an important reason (Li et al., 2020; Yan et al., 2021b). However, current studies have mostly focused on the overall vegetation change and drivers in a region, and less attention has been given to the commonality and variability of drivers among different vegetation types. At the same time, previous studies also assumed a linear relationship between drivers and greenness. Linear regression, residual analysis, and correlation analysis are the methods most commonly used for driver exploration (Chu et al., 2019; Tran et al., 2021; Yan et al., 2021a). However, the response of vegetation to drivers has been proven to be complex and nonlinear (Liu et al., 2016; Myers-Smith et al., 2020). As a classical machine learning algorithm, the random forest (RF) algorithm has the potential to be suitable for exploring such complex relationships (Strobl et al., 2007). It is widely used in the research of biology (An et al., 2019), medicine (Schoning and Hammann, 2018), economics (Barboza et al., 2017) and other fields because it has the characteristics of fast operation speed, it is easy to calculate the nonlinear interaction between variables, and it can reflect the interaction between variables (Breiman, 2001). Meanwhile, relative to general regression analysis, RF models generally do not consider the multivariate covariance problem of potential drivers (Breiman, 2001). Recently, the RF algorithm has been introduced into the study of vegetation dynamics. Qiao et al. (2020) examined the reliability of four methods, including multiple linear regression, generalized additive models, support vector machine, and RF, for driving vegetation change analysis using NDVI time series data from the karst region of southwest China. The results showed that RF had the highest accuracy. Berner and Goetz (2022) used random forest algorithms to identify the most important drivers of greenness change in boreal forest biomes among climate, soil, and topography factors. In these studies, RF algorithms as a machine learning showed high accuracy in predicting vegetation greenness changes and identifying drivers.

The Qinghai-Tibetan Plateau (QTP) is the largest plateau in China and the highest in the world, and is known as the third pole of the world (Teng et al., 2021). It influences the atmospheric circulation in Asia and globally and is the water source for one-fifth of the world’s population, from which the Yangtze, Yellow and Lancang rivers develop and carry more than 150 million sheep units of livestock annually (Cao et al., 2018). The climate and human activities on the QTP have changed significantly over the past decades (Chen et al., 2020). These changes may affect vegetation growth directly or indirectly because multiple ecosystems on the QTP have high vulnerability and are sensitive to climate change. Increased precipitation and warmer temperature are key factors driving vegetation greening (Sun and Qin, 2016; Chen et al., 2020). However, in areas with low and decreasing precipitation, rising temperature can exacerbate drought problems, leading to vegetation browning (Wang et al., 2022). Meanwhile, human activities such as overgrazing and infrastructure construction also have negative impacts on vegetation and soils (Huang et al., 2022). Grassland degradation occurs when the grazing quantity exceeds the carrying capacity. In recent decades, the severe degradation of grasslands on the QTP has created “black soil land”, which has severely damaged ecosystem services such as water conservation and carbon sequestration, and has affected the livelihoods of pastoralists (Dong et al., 2020). From 2000, a series of ecological protection projects have been implemented one after another in areas such as Sanjiangyuan and the Qilian Mountains on the QTP, with a cumulative investment of hundreds of billions of RMB (Shao et al., 2016; Wang et al., 2017; Mu et al., 2022). Due to the diversity of vegetation types on the QTP. To support the success of these projects, it is necessary to monitor vegetation changes and identify key degraded areas on the QTP in recent decades. On this basis, important factors for the browning of different types of vegetation communities need to be identified so that ecological restoration and management measures can be implemented according to local conditions.

In this study, we constructed a time series of vegetation change on the QTP from 2000 to 2021 based on MODIS NDVI data, detected changes in the greenness of the QTP as a whole and in different vegetation communities by trend analysis methods, and further explored the drivers of vegetation browning. The purposes of this study are (1) to identify the vegetation changes on the QTP as a whole and in different vegetation communities from 2000 to 2021, (2) to analyse the drivers of vegetation changes on the QTP as a whole, and (3) to explore the commonalities and differences in the browning of different vegetation types on the QTP. This study can help people further understand the response process of different vegetation communities to natural factors and human activities and provide a scientific basis for the restoration and management of different types of browning vegetation on the QTP.

## Materials and methods

### Study area

**Figure 1:**
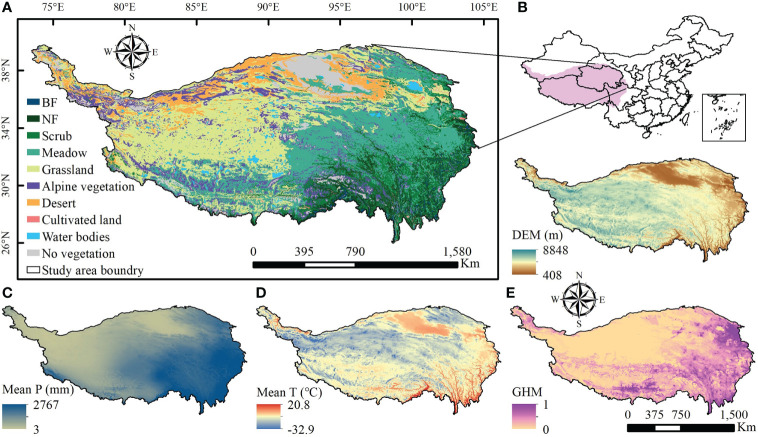
Spatial distribution of vegetation types, topographic and climatic variables, and human activity intensity on the Qinghai-Tibetan Plateau. BF is the broadleaf forest and NF is the needle-leaf forest in **(A)**; in **(B)**, the digital elevation model (DEM) shows elevation of the QTP; in **(C)**, Mean P means mean annual total precipitation from 2000 to 2020; in **(D)**, Mean T represents the mean annual average temperature from 2000 to 2020; and GHM represents the Global Human Modification Index in **(E)**, characterizing the intensity of human activities.

The QTP (25°59′N-39°49′ N, 73°29′ E~ 104°40′ E) is in southwestern China, covering an area of about 254.24×10^4^ km^2^ (**Figure 1**). The average temperature decreases from southeast to northwest. Annual precipitation also shows a gradient of high in the southeast and low in the northwest. Over the last few decades, the QTP has seen a marked increase in temperature and precipitation, with most areas showing marked warming and humidification of the climate. As it straddles several natural zones, the QTP is characterized by a variety of vegetation types. On the southeastern edge of the plateau, forest ecosystem types such as broad-leaved, needle-leaved and mixed forests are developed. In the vast plateau hinterland water ecosystems such as lakes and rivers are formed. Ecosystem types such as meadows, grasslands and deserts, which form at higher altitudes and in colder climates, are some of the largest ecosystem types on the QTP. These natural vegetation ecosystems are distributed in different locations on the QTP and therefore have clear spatial heterogeneity in topography, climate, soil and other conditions. To detect trends of greenness in different vegetation types in response to multiple natural and human impacts, the 1:1 million Chinese vegetation map of the QTP (available from http://westdc.westgis.ac.cn) was divided into seven different vegetation ecosystem zones, including broad-leaved forest (BF), needle-leaved forest (NF), scrub, meadow, grassland, alpine vegetation and desert. Due to the small size of the mixed broad-leaved and needle-leaved forests, they were classified as NF for of statistics and analysis convenience. Glaciers, snow, rocky desert, sandy desert and construction land were combined into no vegetation type.

### Dataset

In this study, NDVI time series, land use data, DEM and environmental variable data are key data (**Table 1**). The NDVI time series data were constructed on the Google Earth Engine (GEE) platform based on the MOD13A2 dataset. The MODIS NDVI product has a resolution of 1000 m and is computed from atmospherically corrected bidirectional surface reflectances that have been masked to remove clouds, cloud shadows, water, and heavy aerosols. We filtered the maximum values of the NDVI for each vegetation growing season (April to September) on the QTP from 2000 to 2021 to characterize the best state of vegetation greenness. The 2020 land use data were obtained from the Resource and Environment Science and Data Center (RESDC) and were used to eliminate built-up land and agricultural land from the vegetated ecosystem areas. Therefore, their classification into seven natural vegetation types was avoid. DEM data were obtained from the NASADEM dataset on the GEE platform at a resolution of 30 m. The environmental variables included precipitation, temperature, soil moisture content, soil pH, soil water content and soil organic matter content. They have been proven to be important for vegetation growth in numerous studies (Feng et al., 2020; Breidenbach et al., 2022). The 1 km monthly precipitation and mean temperature datasets (1901-2020) are from the Tibetan Plateau Data Centre (http://data.tpdc.ac.cn/). Soil data were obtained from the Open land map dataset of the GEE platform with a resolution of 250 m. To consider the impact of human activities on vegetation greenness change, we used the GHM dataset to characterize the intensity of human activities. The dataset considers five main anthropogenic stressors: human settlement, agriculture (cropland, livestock), transport, mining and energy production electrical infrastructure. It characterizes the cumulative intensity of human modification of the land around 2016, ranging from 0 to 1, with a resolution of 1 km. Finally, all data are processed in a raster format, all at a uniform resolution of 1 km.

**Table 1:** Datasets resources in this study.

Dataset	Period	Resolution (m)	Resource
MOD13A2 (NDVI)	2000-2021	1000	Google Earth Engine platform, https://code.earthengine.google.com
1:1 million Chinese vegetation map	2001	–	Hou (2019)
Land use dataset	2020	1000	Resource and Environment Science and Data Center, http://www.resdc.cn
NASADEM dataset	2000	30	NASA JPL (2020)
1-km monthly precipitation dataset for China	2000-2020	1000	Peng (2020)
1-km monthly temperature dataset for China	2000-2020	1000	Peng (2019)
Open land map soil dataset	2017	250	Hengl (2018a); Hengl (2018b); Hengl and Wheeler (2018c); Hengl and Gupta (2019)
Global Human Modification dataset	2016	1000	Kennedy et al. (2019)

### Detection of vegetation greenness change trends

MODIS NDVI data were used to assess the trends and extent of vegetation greenness change on the QTP from 2000 to 2021. This is because a series of ecological conservation and restoration plans and ecological engineering projects have been implemented on the QTP since 2000, and the vegetation condition has improved considerably (Wang et al., 2017; Li et al., 2021a). Meanwhile, this period is the maximum time range for which MODIS NDVI datasets are currently available. The Mann-Kendall test and Sen’s slope trend analysis were combined to identify trends and magnitudes of vegetation greenness change (Wang et al., 2021; Liu et al., 2022). As a nonparametric statistical test, the Mann-Kendall test does not require the data to be normally distributed, only that they are independent (Li et al., 2020). The Mann-Kendall test is also useful for removing noise from time series (Zhou et al., 2020; Zhang et al., 2022). It is widely used to determine whether processes such as climate, hydrology and vegetation greenness change are undergoing natural fluctuations or have a definite trend of change (Wang et al., 2021; Cai et al., 2022). Therefore, the Mann-Kendall test was chosen to determine the significance of trends in vegetation dynamics. For time series *Xi*=(*x*_1_,*x*_2_, ,*x_n_*), the statistic for the Mann-Kendall test is calculated as follow


$$S=\sum_{i=1}^{n-1}\sum_{j=i+1}^{n}\mathrm{sgn}(x_{j}-x_{i})\;i<j\leq n$$

where sgn (*x_j_*-x_i_) is


$$\mathrm{sgn}(x_{j}-x_{i})=\{\begin{array}{c} 1\\ 0\\ -1 \end{array}\;\begin{array}{c} (x_{j}-x_{i})>0\\ (x_{j}-x_{i})=0\\ (x_{j}-x_{i})<0 \end{array}$$

The test statistic Z in the test is defined as follows:


$$Z=\{\begin{array}{c} \left(S-1\right)/\sqrt{V\left(S\right)}\\ 0\\ \left(S+1\right)/\sqrt{V\left(S\right)} \end{array}\;\begin{array}{c} S>0\\ S=0\\ S<0 \end{array}$$

The variance *V(S)* is calculated as follows:


V(S)=n(n−1)(2n+5)/18

where *n* is the length of the time series (in this study, n=22). When *n≥*8, the statistic is approximately normally distributed. The test statistic was then used to test the significance of the trend. In this study, the time series is considered to have a significant trend when Z≥1.96, i.e., at the 95% confidence level.

Based on the significance test, this study further used Sen’s slope trend analysis to detect the direction and magnitude of vegetation greenness change trends on the QTP. The Sen’s slope was calculated as follows:


$$Slope=Median[\left(x_{j}-x_{i}\right)/\left(j-i\right)]\;\forall j>i$$

where the slope value is the trend of change in the time series, *x*i and *x_j_* are the NDVI value of the th and *j*th year. If the slope value is positive, then the vegetation trend is increasing. A negative slope value means that the vegetation greenness change trend is decreasing, indicating a gradual degradation of the vegetation ecosystem. In this study, the vegetation greenness change trends were classified into four types: significant increase (SI), nonsignificant increase (NI), nonsignificant decrease (ND) and significant decrease (SD).

In the process of detecting changes in vegetation greenness, areas with a multiyear NDVI greater than 0.1 are treated as vegetation growth areas, while the rest are considered nonvegetation areas. Moreover, trends in vegetation greenness will be difficult to monitor when NDVI is less than 0.1. Vegetation greenness trend detection was completed on the MATLAB 2020 platform.

### Identification of drivers of vegetation greenness change

The RF classification model was used to identify the main drivers of browning across the QTP and seven vegetation types and the thresholds for the different drivers. A total of 20 variables were used, including climate, topography, soil, and human activity intensity (**Table 2**). Of these, climate variables include the mean, variation, maximum and minimum values (maximum anomalies) of annual total precipitation, annual mean temperature and Summer Warmth Index (SWI) from 2000 to 2020. The SWI represents the sum of the average monthly temperatures greater than 0°C (Keenan and Riley, 2018). It is a better indicator of the environmental heat conditions during the plant growing season than the annual average temperature (Keenan and Riley, 2018; Berner and Goetz, 2022). It is widely used to analyse the effects of climate change on boreal ecosystems, such as tundra (Berner and Goetz, 2022). To investigate the effect of climate change on vegetation greenness, this study also used Sen’s slope trend analysis to calculate the annual trends of total precipitation, average temperature, and SWI. Due to the difficulty of obtaining precipitation and temperature data with 1 km accuracy, the data from 2000 to 2020 were chosen to calculate the climate trends in this study. Topographic factors include elevation (DEM), slope, and aspect. Slope and aspect were calculated on the ArcGIS 10.7 platform based on DEM data. Soil factors include pH, SWC, SOCC, and SBD. Human activity intensity is characterized using the GHM index.

**Table 2:** Potential drivers of vegetation greenness browning and implications.

Variables types	Variables	Description	Units
Climate	Mean P	Mean annual total precipitation from 2000 to 2020	mm
	P slope	Slope of change in annual total precipitation from 2000 to 2020	mm/yr
	Max P	Maximum of annual total precipitation from 2000 to 2020	mm
	Min P	Minimum of annual total precipitation from 2000 to 2020	mm
	Mean SWI	Mean annual SWI from 2000 to 2020	°C
	SWI slope	Slope of change in SWI from 2000 to 2020	°C/yr
	Max SWI	Maximum of annual SWI from 2000 to 2020	°C
	Min SWI	Minimum of annual SWI from 2000 to 2020	°C
	Mean T	Mean annual average temperature from 2000 to 2020	°C
	T slope	Slope of change in annual average temperature from 2000 to 2020	°C/yr
	Max T	Maximum of annual average temperature from 2000 to 2020	°C
	Min T	Minimum of annual average temperature from 2000 to 2020	°C
Topography	DEM	Digital Elevation Model	m
	Slope	Topographic slope (ranged from 0 to 90)	°
	Aspect	Topographic aspect (expressed in positive degrees between 0 and 360 degrees, measured clockwise from north)	°
Soil	pH	Soil pH	–
	SWC	Soil water content	%
	SOCC	Soil organic carbon content	g/kg
	SBD	Soil bulk density	g/cm^3^
Human activity	GHM	Global Human Modification index	–

The RF classification model was used to identify the main drivers of browning across the QTP and seven vegetation types and the thresholds for the different drivers. There are more samples of greening than browning in the QTP and in all seven vegetation types. We used the same number of samples from the greening samples as the browning samples for model training using random selection. 75% of the samples were used for training and 25% of the samples were used for validation. The training accuracy of the RF classification model can be characterized by the out-of-bag error rate. The out-of-bag error can be reduced by adjusting the number of decision trees and the number of evaluation variables per node. The optimal parameters of the model are then determined. In RF classification models, ntree was 100 and mtry was 5. We ranked the importance of the variables to characterize the effect of drivers on vegetation browning. The mean decrease accuracy (MDA) index was used to assess the relative importance of the variables (Strobl et al., 2008). Finally, we identified the main drivers of dominant vegetation browning and plotted the partial dependence of the important drivers. The plot can represent the change of vegetation browning probability with the change in drivers. To avoid errors in a single experiment, we repeated the run 100 times for each random forest classification model, and the average of the results of 100 runs was used as the final result. The overall prediction accuracy of the models can be characterized by receiver operating characteristic (ROC) curves. The ROC curves for 100 repeated runs are shown in the **Supporting Materials**. For the RF classification models, the AUC values of the training and testing data ranged from 0.9 to 0.96.The identification of drivers of vegetation greenness change was performed on the RStduio platform, mainly using packages such as randomForest, raster, and ggplot2.

### Correlation analysis between vegetation greenness and change trends

Spearman correlation coefficient was used to explore the correlation between vegetation greenness and change trends, where vegetation greenness is characterized by the 2021 NDVI values. Spearman correlation coefficient does not require the variables feature normally distributed (Spearman, 1904). It remains applicable when outliers are present or variables feature heavy-tailed distributions (De Winter et al., 2016; Liu et al., 2021b). The analysis was based on the image element scale. Therefore, we analysed more than 640,000 samples with significant changes in vegetation greenness. First, we calculated the relationship between the slopes of vegetation greenness and the NDVI in 2021 on the QTP. Then, we counted the correlation coefficients between the slopes of greening and NDVI for different vegetation types. Finally, we divided the vegetation into greening and browning and explored the correlation between slopes in vegetation greenness and NDVI. Correlation analysis was carried out on the RStudio platform. The absolute value of Spearman’s coefficient ρ was divided into five ranges: 1.0≥|ρ|>0.8 (very strong correlation), 0.8≥|ρ|>0.6 (strong correlation), 0.6≥|ρ|>0.4 (moderate correlation), 0.4≥|ρ|>0.2 (weak correlation), and 0.2≥|ρ|≥0 (very weak correlation).

## Results

### Overall change in greenness of the Qinghai-Tibetan Plateau and seven vegetation types

**Figure 2:**
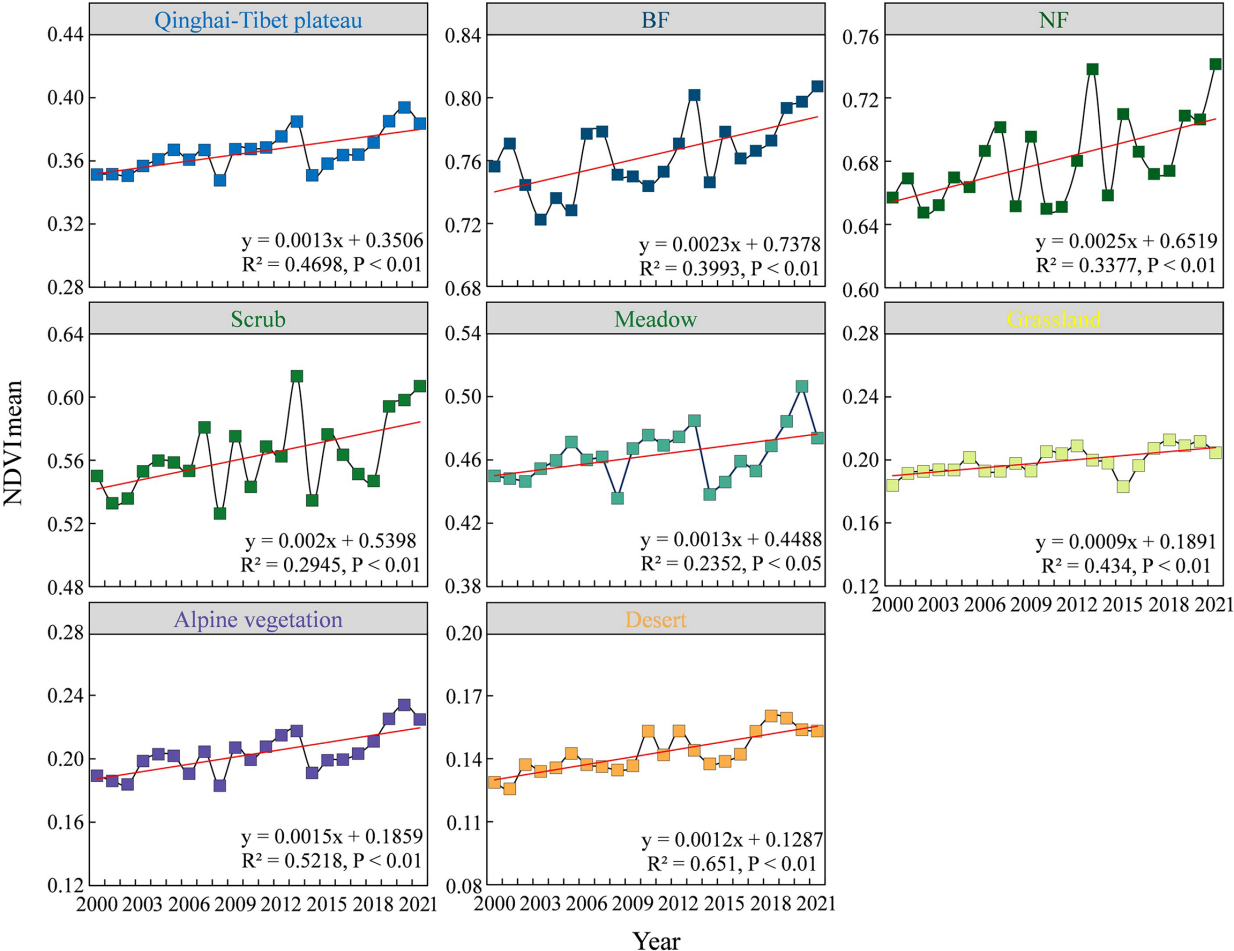
Mean greenness changes of entire Qinghai-Tibetan Plateau and seven vegetation types.

The average greenness trend from 2000 to 2021 shows that the overall greenness of the QTP and the seven different vegetation communities is significantly increasing (**Figure 2**). The average greenness of the QTP is was approximately 0.36. The average greenness of the seven vegetation communities varied clearly, with a size relationship of BF > NF > scrub > meadow > grassland > alpine vegetation > desert. On the QTP, the average greenness increased by approximately 0.0013 per year over 21 years. For the different vegetation communities, the average greenness of BF, NF and scrub increased by more than 0.002 per year, while for meadow, grassland, alpine vegetation and desert, the average greenness increased by approximately 0.001 per year. Therefore, the seven vegetation communities on the QTP show that the higher the vegetation greenness is, the greater the greenness is likely to increase over a 21-year period.

### Spatial distribution and area of vegetation greenness change

**Figure 3:**
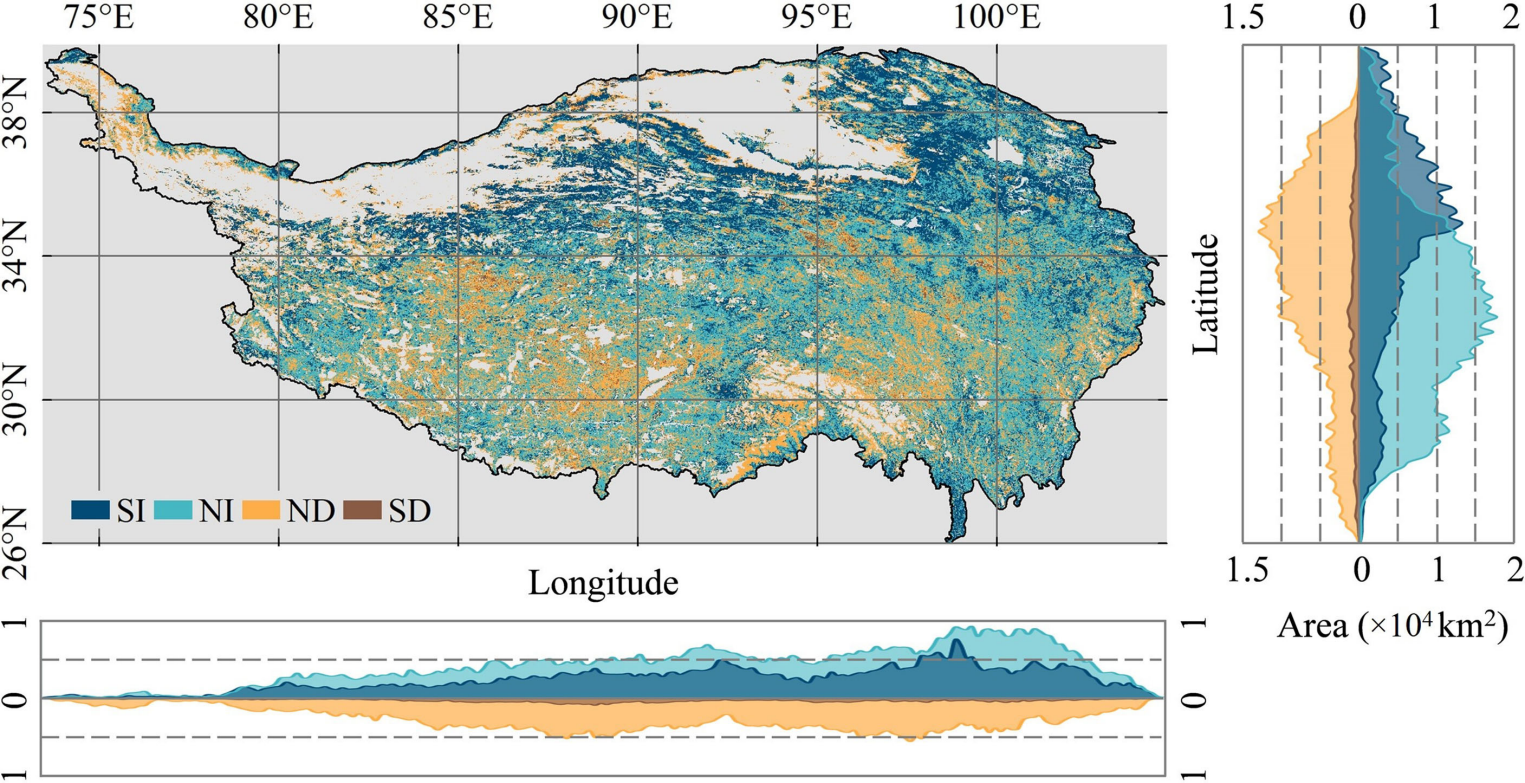
Vegetation greenness changes along longitude and latitude gradients on the Qinghai-Tibetan Plateau.

**Figure 4:**
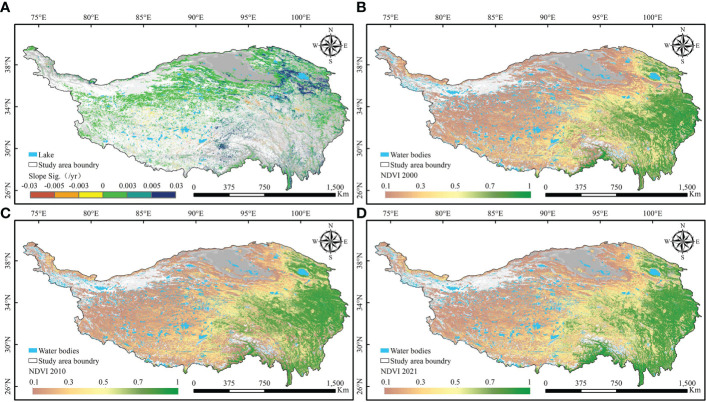
Spatial distribution of vegetation greenness changes on the Qinghai-Tibetan Plateau. **(A)** Significant trends of vegetation greenness. NDVI of the Qinghai-Tibetan Plateau in **(B)** 2000, **(C)** 2010 and **(D)** 2020.

There is a clear spatial aggregation of areas of vegetation greenness change on the QTP (**Figures 3**, **4A**). The northern and northwestern desert areas of the plateau have less vegetation growth. Meanwhile, the snow-covered mountains in the south and the large number of rivers and lakes scattered across the plateau are also without vegetation. The areas of the SI trend in greenness are mainly located in the northern part of the plateau. In both the southwestern and southeastern parts of the plateau, the patches with an SD trend (browning) in greenness are aggregated. In terms of the degree of greenness change, the increase in greenness was most obvious in the area around Qinghai Lake. In the central part of the QTP, vegetation degradation is the most serious. The greenness of vegetation on the entire QTP shows a spatial pattern of “high in the southeast and low in the northwest” (**Figures 4B-D**). In the southeast, there are large areas of forest and scrub with high greenness. The northern and northwestern desert areas of the QTP have less vegetation growth. At the same time, there is no vegetation growth in the high mountains with year-round snow in the south and in the large number of rivers and lakes scattered across the QTP.

**Figure 5:**
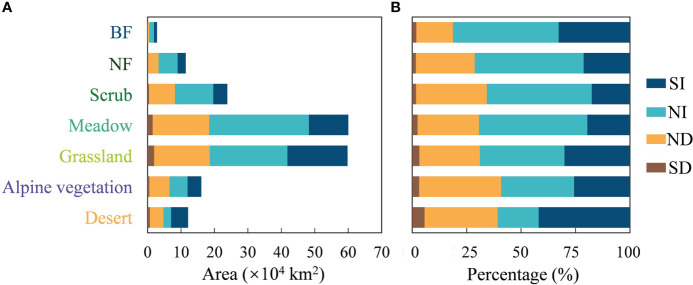
Area statistics of greenness change for seven vegetation types on the Qinghai-Tibet Plateau. **(A)** Area and **(B)** percentages of SI, NI, ND and SD for seven vegetation types.

The total area of the seven vegetation communities is approximately 186.15×10^4^ km^2^. The area statistics for the change in greenness show that the area with an SI in greenness is 46.54×10^4^ km^2^, which is one quarter of the total area of natural vegetation communities (**Figure 5A**). The area with an SD trend in greenness was 5.32×10^4^ km^2^, or 2.86% of the total area of natural vegetation communities. Of the seven different vegetation types, grassland and meadow communities are the largest. They also have the largest area of greening and browning. Grassland has areas of greening and browning of 17.99×10^4^ km^2^ and 1.96×10^4^ km^2^ respectively. For meadow, the areas of greening and browning are 11.79×10^4^ km^2^ and 1.50×10^4^ km^2^. **Figure 5B** shows the composition of greenness change for each vegetation type. Desert has the highest percentage of greening at 41.92%. From BF to desert, the percentage of area browning tends to increase, with desert having the highest browning percentage at 5.64%. However, BF, NF and scrub all have less than 2%.

### Drivers of vegetation greenness browning on the Qinghai-Tibet Plateau

**Figure 6:**
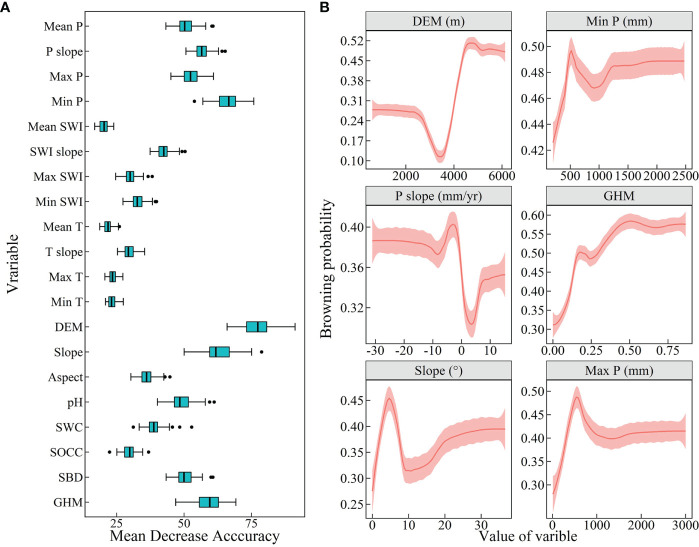
Feature importance and partial bias dependence maps of vegetation greenness browning on the Qinghai-Tibet Plateau. **(A)** Feature importance of 20 variables. **(B)** The top 6 drivers of feature importance were selected to plot their partial bias dependencies. The order of importance, from highest to lowest, is DEM, Min P, P slope, GHM, Slope and Max P. The light red area indicates the 95% confidence interval.

This study used the RF model to assess the degree to which vegetation greenness browning was associated with climate, topography, soils, and human activities over 21 years. This led to the identification of key variables that influence vegetation degradation. The six variables with the highest feature importance were selected as important drivers. For the entire QTP, topography, precipitation, and the intensity of human activities were most important (**Figure 6**). DEM, the minimum annual total precipitation, ranked highest in importance, followed by change in mean annual total precipitation and intensity of human activities (GHM) and topographic slope and mean annual maximum precipitation. The bias dependence plot shows that areas with high altitude, reduced annual precipitation and high intensity of human activities are more likely to brown. The probability of vegetation browning was higher when the minimum and maximum of annual total precipitation were approximately 500 mm. In addition, meadows and grasslands are mainly distributed in such places. The vegetation has a higher browning probability in areas with slopes approximately 5° and higher. Conversely, based on the browning probability, we can also know the greening probability of vegetation. That is, greening is most likely to occur in areas with low to medium altitudes, increased average annual precipitation, and low intensities of human activities.

### Drivers of greenness browning for different vegetation communities

**Figure 7:**
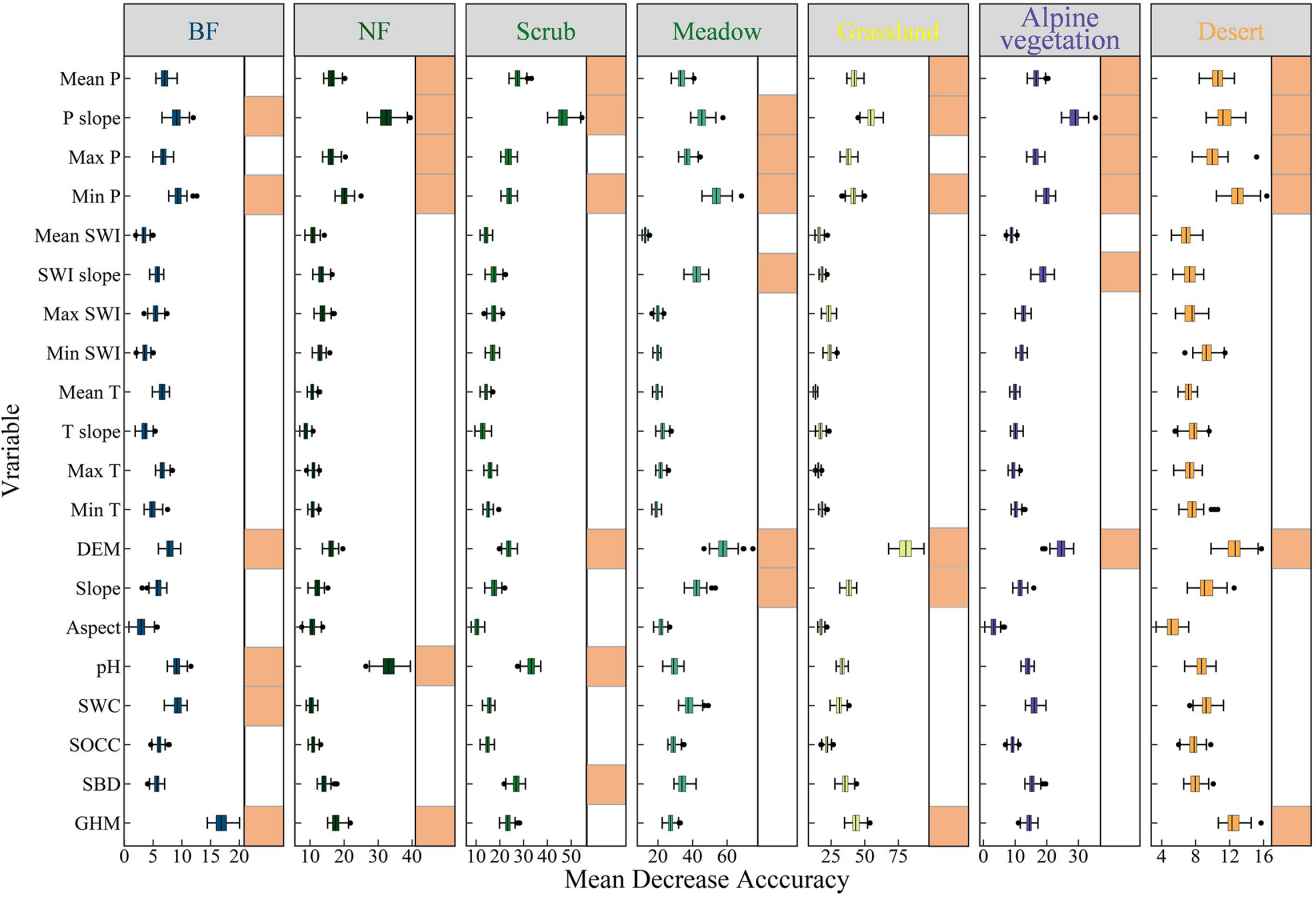
Feature importance of seven vegetation types. The orange square after a variable is used to indicate that the variable is ranked in the top 6 out of 20 variables in terms of feature importance.

The results of the variable importance of the RF classification model indicate that changes in precipitation are an important driver affecting the browning of the seven vegetation types on the QTP. **Figure 7** shows that for all seven vegetation types, the change in annual total precipitation from 2000 to 2020 is one of the six most important variables. For different vegetation types, drought may be an important cause of the browning of vegetation greenness in all of them. Moreover, the mean and maximum anomalies of precipitation are also important predictors of vegetation greenness browning. This further reflects the sensitivity of vegetation to precipitation changes on the QTP. For SWI and temperature variables, only the SWI trend is important in predicting greenness browning in both meadow and alpine areas. The two vegetation types may be more sensitive to temperature changes. Among the topographic variables, elevation is an important variable in predicting greenness browning for all six vegetation types except NF. For meadow and grassland, slope is also an important influencing factor. The soil factor was only more important in the three greenest vegetation types, BF, NF and scrub. The human activity intensity factor is less important only in meadow and grassland. For all seven different vegetation types, precipitation indicators are the most important, while there are some differences in the importance of SWI, temperature, soil and human activity intensity indicators in predicting the greenness browning of different vegetation types.

## Discussion

### Common and different drivers of greenness browning in vegetation types

Generally, the natural vegetation shows a greening trend on the QTP from 2000 to 2021. However, there are still a few areas where vegetation degradation has occurred, which is consistent with previous studies (Peng et al., 2012; Liu et al., 2021a). For example, Liu et al. (2022) found that the proportion of significant browning image elements was 2.6% and the proportion of significant greening image elements was 21.7% on the QTP from 2000 to 2015. In our study, the area of significantly browning accounted for 2.86% of the total vegetation area, and the area of significantly greening was 25%. In previous studies, NDVI decrease areas were also monitored mainly in Qumalai and Zhiduo counties in the central part of the QTP, and in Nagqu and Dangxiong counties in the south, which are the same as the results of this study (Pan et al., 2017; Zhang et al., 2021). Most of these vegetation degradation areas have fragile ecological background conditions and may have been degraded by human activities over time (Huang et al., 2022). For example, Qumalai County in Qinghai Province has a short growing period of meadow, and overgrazing, rodent and pest infestation, and human damage have caused serious degradation of meadows (Zhou et al., 2021).

The drivers of vegetation greenness browning can be divided into natural factors and human activities. The different vegetation types on the QTP are distributed in distinct zones. The cause of browning of different vegetation types depends on which factor or combination of factors is the main local limiting factor (Lehnert et al., 2016). This study found that precipitation, topographic factors and human activities contributed the most to vegetation browning. These results are generally consistent with previous studies (Sun et al., 2013; Jiao et al., 2021; Huang et al., 2022). The positive correlation between precipitation and vegetation greenness was confirmed globally, including on the QTP. Precipitation tended to increase in the eastern and northern parts of the QTP, while in the southern and central parts, precipitation tended to decrease (**Supplementary Figure 1**). The browning of vegetation in these areas can be explained by the decrease in precipitation. Especially in areas with lower precipitation, vegetation is more sensitive to the decrease in precipitation. Some studies emphasized the important role of temperature changes in QTP in vegetation change (Chen et al., 2020). In contrast, our study found that only in meadows, SWI slope was an important drivers of greenness browning. This may be due to the spatially asynchronous variability of hydrothermal conditions on the QTP. Except for the northern and western parts, the QTP showed a trend of increasing temperature. The increase in temperature is most evident in areas where meadows are distributed. When temperature increases while precipitation remains or decreases, vegetation browning may occur. With increasing elevation, the sensitivity of vegetation to climate change increases (Wei et al., 2022). The risk of vegetation browning increases with increased drought or anthropogenic disturbance. The probability of greenness browning of NF and scrubs is also greater when the soil pH is weakly alkaline (pH>7). The human activity intensity is an important driver in BF, NF, grassland and desert. Urbanization and other infrastructure construction activities may be an important cause of browning in BF, NF and Desert. The browning of grassland may be due to the high intensity of grazing in the southern QTP.

### The relationship between vegetation greenness and change trend

**Figure 8:**
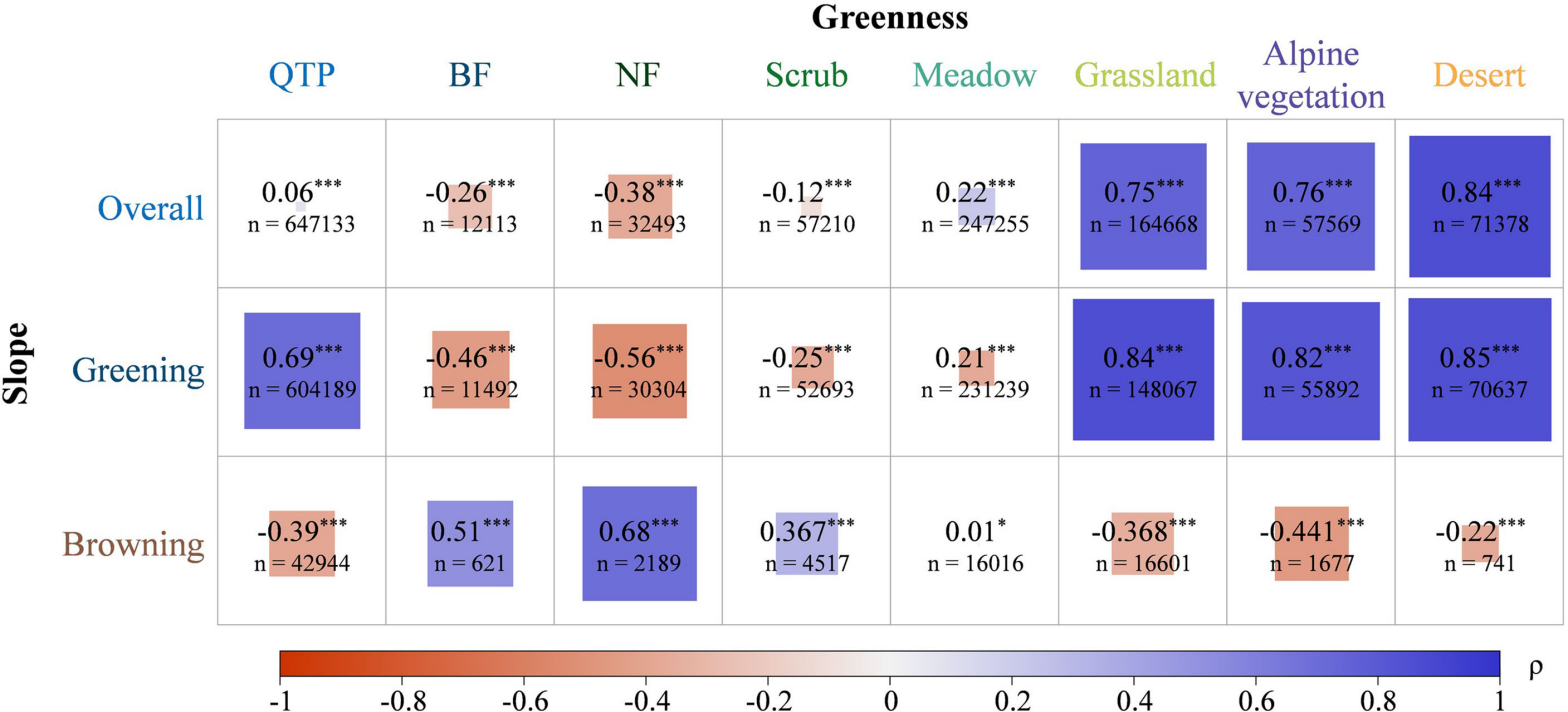
Spearman coefficients between vegetation greenness and change trend. Slope of greening is greater than 0, and slope of browning is less than 0. *** and * represent significant at 99% and 90% confidence levels, respectively. The absolute value of Spearman’s coefficient ρ is divided into five ranges: 1.0≥**|p|**>0.8 (very strong correlation), 0.8≥**|p|**>0.6 (strong correlation), 0.6≥**|p|**>0.4 (moderate correlation), 0.4≥**|p|**>0.2 (weak correlation), and 0.2≥**|p|**≥0 (very weak correlation).

The results show that for the seven vegetation types on the QTP, the vegetation types with higher greenness seemed to increase more. To further investigate the relationship between greenness magnitude and greenness trends, we calculated Spearman correlation coefficient between the two (**Figure 8**). Spearman coefficients between greening and browning trends and vegetation greenness were calculated to clarify the effect of greenness on greening and browning in different vegetation types. During the analysis, we only selected the image elements with significant changes in greenness. From the entire QTP, a very weak positive correlation is shown between greenness and slope. When the greening and browning parts were considered separately, the slope of greening and greenness showed a strong positive correlation, while the slope of browning and greenness showed a weak negative correlation. For both forest communities, the slope of greening and greenness showed a moderate negative correlation, while the slope of browning and greenness showed moderate and strong positive correlations. That is, the slope of both greening and browning tended to be close to 0 in areas with greater NDVI. Vegetation with lower NDVI may be more susceptible to greening and browning. For scrub communities, scrubs with lower greenness were more prone to browning. In contrast, there was a significant strong correlation between the greenness and slope of greening of grasslands, i.e., grasslands with higher greenness were also more likely to be greened. In meadow communities, the correlation between greenness and slope was very weak. For both vegetation types, alpine vegetation and desert, similar relationships with grasslands were shown, where the higher the greenness the more susceptible to greening. Ecosystem stability varies significantly among different vegetation types (Kang et al., 2022). Overall, in BFs, NFs and scrubs with higher NDVIs, areas with higher greenness tended to be stable, and areas with lower greenness were prone to greening and browning. This may be because, more vegetation greenness is, the more stable the vegetation condition is, as the area tends to be in the climax community in forests. Meanwhile, some studies reported a significant increase in the stability of forest community productivity with increasing species richness (Schnabel et al., 2021). While in grasslands, alpine vegetations and deserts, areas with high greenness are more prone to greening.

### Natural vegetation restoration recommendations

The causes of vegetation degradation are diverse. This study identified the drivers of browning in different vegetation communities on the QTP, mainly including precipitation, topography, and human activities. We recommend appropriate restoration measures by considering the degree of browning and the dominant drivers. Depending on the degree of vegetation browning, different levels of anthropogenic restoration measures should be taken to maximize the resilience of natural ecosystems (Cao et al., 2018; Wen et al., 2019). For example, for lightly browned vegetation, natural restoration through enhanced management is generally all that is needed to achieve restoration goals. For vegetation with moderate browning, biological measures are needed to assist in restoration. For heavily browned vegetation, ecological restoration by physical modification is needed (Teng et al., 2020). Human activity intensity was an important driver for BF and desert browning. For these two vegetation types, human disturbance should be excluded first, and the appropriate measures should be taken according to the degree of browning. For vegetation sensitive to precipitation changes and other natural factors, browning scale and degree need to be to be monitored more closely, and restoration measures should be taken at the appropriate time to prevent browning from intensifying. In conclusion, we support the adoption of artificially guided vegetation restoration measures according to local conditions.

### Limitations and perspectives

In our study, there are several limitations that should be further considered and improved upon in future studies. First, considering the availability of data, we selected 20 potential drivers of greenness change from four aspects: climate, topography, soil, and human activities. To ensure heterogeneity in data resolution, all data in our study were scaled to 1 km. The applicability of our findings at other scales may need to be further explored due to scale effects. Additionally, extreme climate events are considered to have important impacts on ecosystems such as grasslands (Hoover et al., 2022). The inclusion of extreme climate data in the study could be considered in the future. Second, our study reveals the commonality and specificity of the drivers of vegetation browning on the QTP. This is beneficial for increasing the understanding of vegetation change and thus supporting ecosystem management and restoration efforts on the QTP and in other regions. We will try to distinguish the spatial heterogeneity of drivers and differences in the response of different vegetation communities, thus providing scientific support for the implementation of ecological projects.

## Conclusion

This study calculated the vegetation change trends on the QTP from 2000 to 2021 based on the MODIS NDVI dataset and determined the spatial distribution of vegetation greening and browning areas. The RF model was used to identify the drivers of browning on the QTP as a whole and for seven vegetation types. This study found that the greenness of the entire QTP and the seven different vegetation types showed an overall increasing trend over the past 20 years. The NDVI of the Qinghai-Tibetan Plateau increased by an average of 0.0013 per year. The greening areas are mainly concentrated in the northern part of the Qinghai-Tibetan Plateau, accounting for 25% of the natural vegetation area. The browning patches were mainly distributed in the central and south parts of the QTP, covering 2.86% of the natural vegetation area. Meanwhile, precipitation, topography and human activities were identified as the important drivers of vegetation browning on the QTP. For different vegetation types, we also identified the variability in browning drivers. This is reflected in the fact that the main drivers of the seven vegetation types are not exactly the same, as well as their different rankings of importance. Our results show that there are differences in the drivers of browning among different vegetation types and identify the main drivers of different vegetation types, providing a scientific basis for ecosystem management and restoration on the QTP.

## Data availability statement

The original contributions presented in the study are included in the article/**Supplementary Material**. Further inquiries can be directed to the corresponding author.

## Author contributions

HW: conceptualization, methodology, data curation, formal analysis and writing - original draft. JZ: writing - review & editing, resources, supervision. CW, WL, ZY, HL, CB: data curation, formal analysis, writing - review & editing. All authors contributed to the article and approved the submitted version.

## Funding

This study was supported by the Second Scientific Expedition to the Qinghai-Tibet Plateau (Grant No. 2019QZKK0405-05) and the State Key Program of the National Natural Science Foundation of China (Grant No. 72033005).

## Conflict of interest

The authors declare that the research was conducted in the absence of any commercial or financial relationships that could be construed as a potential conflict of interest.

## Publisher’s note

All claims expressed in this article are solely those of the authors and do not necessarily represent those of their affiliated organizations, or those of the publisher, the editors and the reviewers. Any product that may be evaluated in this article, or claim that may be made by its manufacturer, is not guaranteed or endorsed by the publisher.
