# Supplementary material for: Greening or browning? The macro variation and drivers of different vegetation types on the Qinghai-Tibetan Plateau from 2000 to 2021
Source: Front Plant Sci. 2022 Oct 26;13:1045290. doi: 10.3389/fpls.2022.1045290 (PMC9643839; doi:10.3389/fpls.2022.1045290)
Supplement: Supplementary file 1 [file DataSheet_1.docx]

**Supplementary material for**

**Greening or browning? The macro variation and drivers of different vegetation types on the Qinghai-Tibetan Plateau from 2000 to 2021**

**Huihui Wang^1^, Jinyan Zhan^1*^, Chao Wang^2^, Wei Liu^3^, Zheng Yang^1^, Huizi Liu^1^, Chunyue Bai^1^**

^1^State Key Laboratory of Water Environment Simulation, School of Environment, Beijing Normal University, Beijing 100875, China

^2^School of Labor Economics, Capital University of Economics and Business, Beijing 100070, China

^3^College of Geography and Environment, Shandong Normal University, Jinan 230358, China

***Correspondence:**Jinyan Zhan
zhanjy@bnu.edu.cn

**Totally 2 pages include 2 figures**

**Supplementary Figure 1.** Spatial distribution of 20 potential drivers of vegetation browning on the Qinghai-Tibetan plateau.

**Supplementary Figure 2.** ROC curves for RF model simulations.


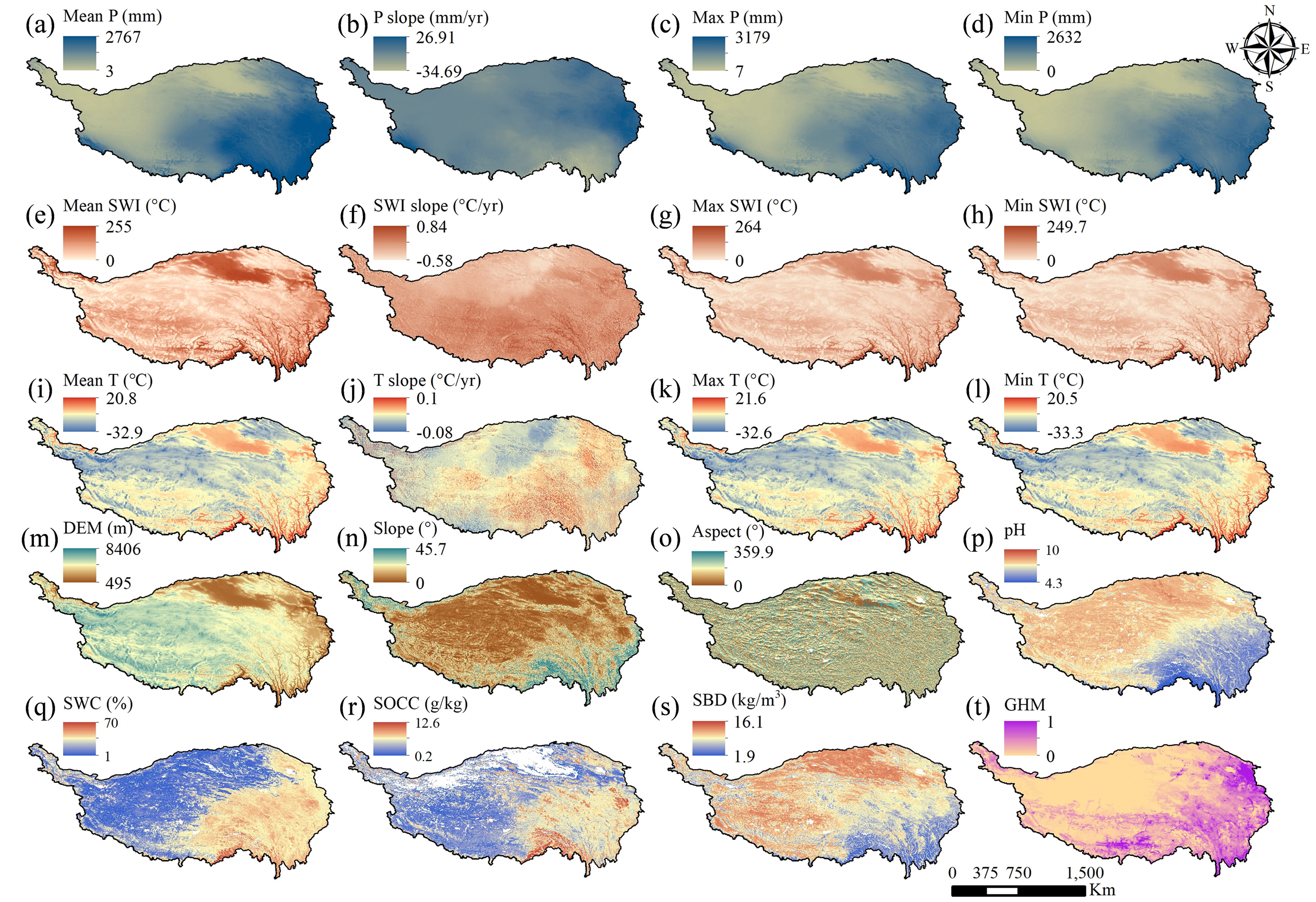


**Supplementary Figure 1.** Spatial distribution of 20 potential drivers of vegetation browning on the Qinghai-Tibetan plateau.


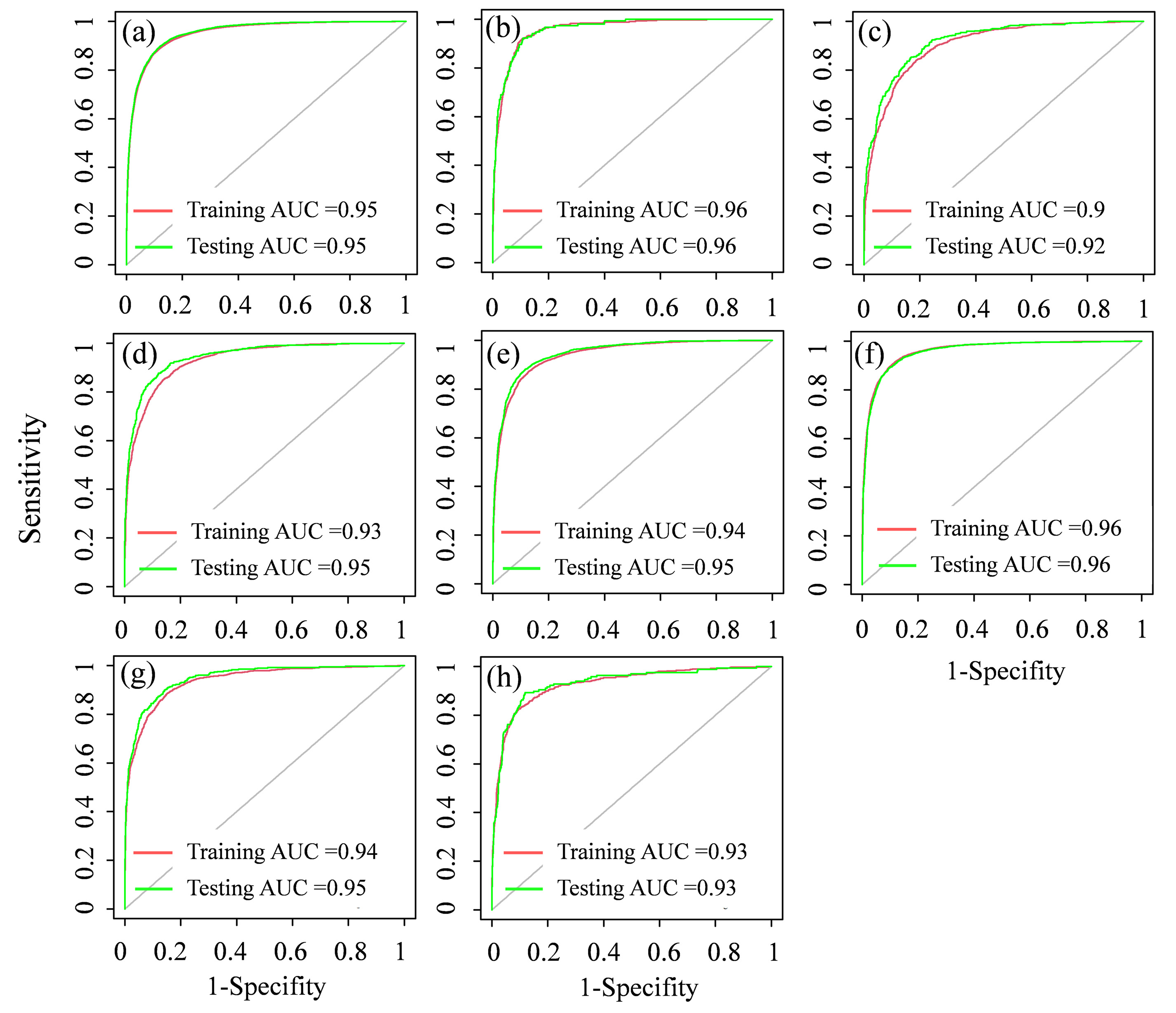


**Supplementary Figure 2.** ROC curves for RF model simulations. Supplementary Figure 2a-g represent Qinghai-Tibetan plateau, BF, NF, Scrub, Meadow, Grassland, Alpine vegetation, Desert respectively)
